# Severe Corticosteroid-Induced Mania With Psychotic Features in an HIV-Positive Patient: A Case Report

**DOI:** 10.7759/cureus.99486

**Published:** 2025-12-17

**Authors:** Salmane El Kodsi, Jalal Elouadoudi, Mehdi Denani, Mohammed Amine Alfa, Mahmoud Amine Laffinti

**Affiliations:** 1 Department of Psychiatry, Avicenna Military Hospital, Marrakech, MAR

**Keywords:** acute mania, bipolar disorder (bd), corticosteroids, haloperidol, hiv positive

## Abstract

Corticosteroids are widely used to treat numerous inflammatory conditions but are associated with a significant risk of psychiatric adverse effects, including mood disturbances, psychosis, and mania. These reactions may be challenging to recognize in medically complex patients. We report the case of a 40-year-old woman who developed an acute manic episode with mood-congruent psychotic features three days after initiation of intravenous methylprednisolone (80 mg/day) for hypoxemic respiratory failure, later diagnosed as probable *Pneumocystis jirovecii* pneumonia in the context of newly diagnosed HIV infection. The clinical picture was marked by euphoric mood, behavioral disinhibition, pressured speech, increased goal-directed activity, reduced need for sleep, and mood-congruent delusions and hallucinations. The differential diagnosis included steroid-induced psychosis versus an organic psychiatric disorder secondary to HIV encephalopathy or a CNS opportunistic infection. Brain MRI revealed no acute intracranial abnormalities, and additional investigations were unremarkable. Haloperidol was introduced to manage the acute phase, while corticosteroids were rapidly tapered and then discontinued. A rapid and complete resolution of manic and psychotic symptoms was observed following steroid withdrawal, with sustained remission despite subsequent tapering and cessation of antipsychotic treatment. This case illustrates a typical presentation of corticosteroid-induced bipolar and related disorder and underscores the need to consider this iatrogenic etiology even in the presence of significant medical confounders such as a new HIV diagnosis. It also highlights the diagnostic value of symptom resolution after drug discontinuation in supporting causality.

## Introduction

Since the 1950s, glucocorticoids have been widely used to treat a variety of pathologies due to their potent anti-inflammatory and immunosuppressive properties [1,2]. They remain essential in the management of severe asthma and chronic obstructive pulmonary disease exacerbations, systemic lupus erythematosus, rheumatoid arthritis, and inflammatory bowel diseases. However, research has shown that their use is associated with a significant risk of psychiatric side effects, such as anxiety, mood disorders, psychosis, and mania [3,4]. These complications are reported in a small but notable proportion of patients, with higher doses carrying a greater risk. These psychiatric disorders present a complex diagnostic challenge, particularly in patients with multiple medical comorbidities where symptoms may be confounded by the underlying illness, such as a CNS infection or an inflammatory process [1]. We report a case of acute corticosteroid‑induced bipolar and related disorder to illustrate this major iatrogenic complication and the diagnostic reasoning required in a complex clinical setting.

## Case presentation

We report the case of a 40-year-old woman, married and mother of three children, with no documented personal or family psychiatric history. Her medical history was notable for rosacea treated one year before.

The patient was admitted to the internal medicine department for acute respiratory failure. The somatic history began one year earlier with intermittent diarrhea, followed by significant weight loss and exertional dyspnea (Stage 2 of the New York Heart Association or NYHA) over the preceding nine months. Her condition acutely worsened two days before admission with the onset of high fever.

**Figure 1:**
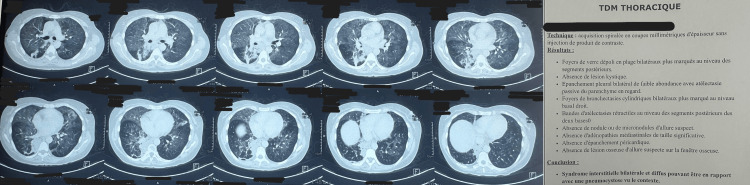
CT images showing diffuse bilateral ground‑glass opacities (left panel) and the corresponding radiology report (right panel).

Upon admission, the clinical examination found a febrile patient, tachycardic at 140 bpm, with hypoxia (oxygen saturation at 80% on room air, improving to 95% on 5 L/min of oxygen). Pulmonary auscultation revealed bilateral crackles. Laboratory findings showed profound lymphopenia, microcytic anemia, elevated CRP at 216 mg/L, and procalcitonin at 3.58 ng/mL. A thoraco-abdomino-pelvic CT scan revealed diffuse bilateral ground-glass opacities, as shown in Figure 1.

The etiological investigation led to the diagnosis of a new HIV infection (confirmed by positive viral load). The respiratory presentation was diagnosed as probable *Pneumocystis jirovecii* pneumonia, constituting the revealing manifestation of the HIV infection.

Somatic management was initiated with Tavanic (levofloxacin) and intravenous corticosteroid therapy (methylprednisolone 80 mg/day) starting on Day 1 (D1) for the respiratory failure, leading to rapid respiratory improvement.

However, on D3 of hospitalization, the patient exhibited a rapid and profound alteration in her mental state, marking the onset of a classic manic episode. This was characterized by a distinct period of abnormally and persistently elevated, expansive, and euphoric mood. This mood disturbance was accompanied by a significant increase in goal-directed activity and energy. The psychiatric examination revealed a high-flow, pressured speech (logorrhea), making coherent conversation difficult. We noted a marked behavioral disinhibition and an overly familiar behavior with staff and other patients. Concurrently, she showed clear distractibility, with her attention easily drawn to irrelevant external stimuli. 

Faced with this syndrome, a cerebral MRI was performed, which showed no acute intracranial abnormalities, helping to rule out an acute CNS infectious or encephalopathic process. Complementary blood tests were also unremarkable.

After a psychiatric consultation and a multidisciplinary team discussion, the syndrome was attributed to the high-dose corticosteroid therapy (substance/medication-induced bipolar and related disorder per Diagnostic and Statistical Manual of Mental Disorders, Fifth Edition or DSM-5 criteria). A decision was made to progressively taper the methylprednisolone (at a rate of 10 mg/day) and to introduce symptomatic treatment with risperidone (2 mg/day) and levomepromazine (25 mg/day). 

Despite this intervention, on D7, the patient's psychiatric state escalated into a severe manic episode with psychotic features. Her agitation evolved into severe psychomotor agitation, with overt aggressiveness. The psychotic phenomena became prominent: the patient reported both auditory and visual hallucinations. Critically, these hallucinations were mood-congruent, involving grandiose themes. Her thought process was highly disorganized, consistent with a flight of ideas, and she developed total insomnia, reporting no need for sleep. 

Given this severe worsening and the failure of the initial strategy, the corticosteroid therapy was completely discontinued. The therapeutic protocol was revised to haloperidol drops (20 drops twice daily) under strict monitoring.

A marked improvement in the entire psychiatric symptomatology was observed 48 hours after the total cessation of corticosteroids. This remission was sustained even as the haloperidol dosage was progressively tapered (down to 10 drops/day) and eventually stopped. The patient was discharged on D14.

**Figure 2:**
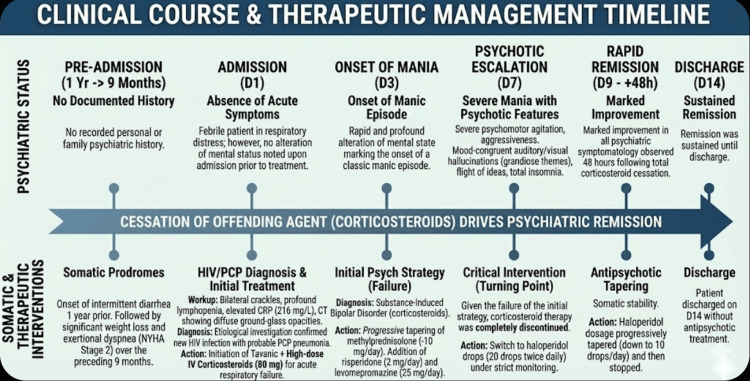
Temporal correlation between corticosteroid therapy and psychiatric course onset, management, and resolution.

A detailed timeline of the patient’s clinical course and therapeutic management is shown in Figure 2.

## Discussion

Clinical analysis and diagnostic considerations

Psychiatric complications of systemic corticosteroid therapy are well documented. In a retrospective review, Warrington and Bostwick reported severe reactions in approximately 5.7% of exposed patients and mild-to-moderate reactions in approximately 28% [5]. In a series of 55 published cases, Kenna et al. found that hypomanic or manic episodes accounted for 54.5% of presentations, depressive episodes for 23.6%, and delirium for 20% [6]. Drawing on data from the Boston Collaborative Drug Surveillance Program, Hara et al. highlighted a clear dose-response relationship: psychiatric adverse effects occurred in 1.3% of patients receiving ≤40 mg/day of prednisone equivalent, 4.6% at 41-80 mg/day, and 18.4% at doses above 80 mg/day [7]. Across these studies, manic or mixed states consistently represent roughly one-third of corticosteroid-related psychiatric events [1-3].

In this context, our patient exhibited a clinical picture consistent with an acute manic episode with mood-congruent psychotic features. The psychiatric assessment revealed a prominently elevated mood, marked behavioral disinhibition, accelerated thought processes with flight of ideas, pressured speech, a pronounced reduction in the need for sleep, and significant psychomotor agitation, alongside grandiose delusional ideas and mood-congruent hallucinations. Symptom severity was quantified using the Young Mania Rating Scale (YMRS) [8]. On D3, after initiation of intravenous methylprednisolone at 80 mg/day, the YMRS score was 12, compatible with a mild manic episode. By D7, amidst a clear escalation of agitation, disinhibition, and delusional ideation, the YMRS had increased to 32, indicating a severe manic episode. It was at this point that the treatment strategy was reassessed, leading to the withdrawal of corticosteroids and the introduction of haloperidol.

The temporal profile and severity trajectory observed in our case closely mirror those described in the literature. Hara et al. reported a 30-year-old woman who developed a severe manic episode a few days after a methylprednisolone pulse, with a YMRS score of 41, requiring antipsychotic treatment [7]. Fatheddine et al. described a young woman treated for acute disseminated encephalomyelitis who developed a manic episode, characterized by agitation, logorrhea, disinhibition, and insomnia, in close temporal association with corticosteroid dose adjustment, followed by improvement after dose reduction and initiation of olanzapine [1]. Our observation shares several key features with these reports, including female sex, exposure to high-dose corticosteroids, short latency between steroid initiation and symptom onset, and a typical manic phenotype, and therefore fits well within the spectrum of corticosteroid-induced manic states [1-3,9].

The new diagnosis of HIV infection with *Pneumocystis jirovecii* pneumonia broadens the differential diagnosis. In people living with HIV, an acute manic or psychotic episode necessitates consideration of HIV-associated encephalopathy, opportunistic CNS infections, metabolic or toxic encephalopathy, and primary mood or psychotic disorders. In our patient, several converging elements supported the diagnosis of a steroid-induced mood disorder. First, a tight temporal relationship existed between the initiation of methylprednisolone and both the onset and subsequent worsening of manic symptoms (from D3 to D7), consistent with the peak risk window around the third to fourth day of treatment reported by Hara et al. and Bachu et al. [7,9]. Second, there was no frank confusional state or focal neurological deficit. Third, brain MRI revealed no acute lesions, and routine laboratory investigations showed no metabolic, infectious, or toxic abnormalities that could account for the acute psychiatric presentation. Finally, there was a rapid and complete remission of manic and psychotic symptoms following corticosteroid tapering and antipsychotic treatment, with sustained clinical stability despite subsequent dose reduction of haloperidol. This evolution is highly characteristic of corticosteroid-induced psychiatric syndromes [6,9].

The possibility of a first manic episode as part of bipolar I disorder must also be addressed. Several authors have suggested that corticosteroids may unmask an underlying bipolar diathesis, with some patients subsequently experiencing spontaneous mood episodes in the absence of further steroid exposure [6,7]. In the case described by Hara et al., the patient had a history of subthreshold mood fluctuations and later developed episodes consistent with a fully syndromal bipolar disorder [7]. By contrast, our patient had no prior history of syndromal mood episodes, no documented family history of bipolar disorder, and the index episode exclusively in the context of acute exposure to high-dose corticosteroids. Moreover, no recurrent mood episode was observed during the available follow-up period after discontinuation of corticosteroid therapy. Taken together, these elements argue in favor of a diagnosis of corticosteroid-induced bipolar and related disorder rather than bipolar I disorder while acknowledging that only longer-term follow-up can definitively exclude the emergence of a primary bipolar illness.

Management and the role of haloperidol in an atypical context

Clinical management relied on a coordinated, dual-track strategy, rapid tapering of systemic corticosteroids, and sequential adjustment of the antipsychotic regimen, with the goal of achieving rapid stabilization of a severe manic episode in the context of newly diagnosed HIV infection and hypoxemic *Pneumocystis jirovecii* pneumonia.

In accordance with established recommendations, a conservative first-line approach was implemented. Risperidone 2 mg/day was initiated, combined with levomepromazine 25 mg at night to address insomnia, anxiety, and psychomotor agitation. At this stage, manic symptoms remained moderate (YMRS = 12 on D3). This strategy is consistent with literature supporting the use of second-generation antipsychotics such as risperidone or olanzapine in early presentations of corticosteroid-induced mood or psychotic disorders, particularly among patients with significant medical comorbidities [6,10].

However, the patient’s evolution into a severe manic episode with mood-congruent psychotic features (YMRS = 32 on D7), despite adequate adherence and careful titration, constituted a partial non-response to the risperidone-levomepromazine regimen. This abrupt clinical escalation reflects the unpredictable course of corticosteroid-induced mania, a pattern repeatedly described in the literature, where atypical antipsychotics may yield incomplete or delayed effects in severe cases [6,10].

Given the combination of psychiatric severity, somatic instability, and the need for rapid behavioral control, a therapeutic switch to haloperidol was performed. The liquid formulation (20 drops twice daily, ≈4 mg/day) was selected to enable precise titration and ensure reliable absorption in an acute setting while maintaining low-dose levomepromazine and continuing corticosteroid tapering.

Several publications support the use of haloperidol as an effective second-line option in corticosteroid-induced psychiatric syndromes. In a review of 13 cases, Huynh and Reinert reported that haloperidol was among the most frequently employed agents, with doses such as 5 mg IV followed by 2 mg twice daily orally or 2.5 mg three times daily, leading to complete clinical remission once corticosteroids were tapered [11]. The Palliative Care Network of Wisconsin likewise notes that low-dose haloperidol (0.5-1 mg/day) is effective for resolving corticosteroid-induced psychosis or mania, with efficacy comparable to risperidone and olanzapine [12].

Most notably, Bachu et al. described a patient with corticosteroid-induced psychosis who had failed or refused risperidone and was successfully stabilized on liquid haloperidol 2 mg twice daily, equivalent to the regimen used in our patient [9]. Additional case reports, such as those presented by Canessa-Muñoz et al., confirm that antipsychotic treatment, typical or atypical, combined with corticosteroid discontinuation generally leads to rapid improvement and favorable outcomes [13].

## Conclusions

This single case illustrates how severe manic and psychotic symptoms can emerge in close temporal association with systemic corticosteroid therapy in an HIV-positive patient, and resolve after steroid discontinuation and targeted psychiatric treatment. The observation underscores the importance of systematically considering iatrogenic causes, including corticosteroid-induced bipolar and related disorder, when evaluating acute mood or psychotic episodes in medically complex patients. In our patient, low-dose liquid haloperidol (4 mg/day, then gradually tapered) proved effective and well-tolerated after an insufficient response to an initial atypical antipsychotic, allowing rapid behavioral control without significant extrapyramidal symptoms or QTc prolongation. Given the single-case nature of this report and the limited follow-up, these findings must be interpreted with caution and are best viewed as an illustrative example that may help clinicians balance the psychiatric risks of corticosteroids against their somatic benefits and guide antipsychotic choices in similar situations.
